# The prognostic value of resting-state EEG in acute post-traumatic unresponsive states

**DOI:** 10.1093/braincomms/fcab017

**Published:** 2021-03-17

**Authors:** Alice O’Donnell, Ruth Pauli, Leah Banellis, Rodika Sokoliuk, Tom Hayton, Steve Sturman, Tonny Veenith, Kamal M Yakoub, Antonio Belli, Srivas Chennu, Damian Cruse

**Affiliations:** 1 Birmingham Medical School, University of Birmingham, Edgbaston B15 2TT, UK; 2 Centre for Human Brain Health, University of Birmingham, Edgbaston B15 2TT, UK; 3 School of Psychology, University of Birmingham, Edgbaston B15 2TT, UK; 4 National Institute for Health Research Surgical Reconstruction and Microbiology Research Centre, Birmingham B15 2TH, UK; 5 Birmingham Acute Care Research Group, Institute of Inflammation and Ageing, University of Birmingham, Edgbaston B15 2TT, UK; 6 School of Computing, University of Kent, Canterbury CT2 7NZ, UK

**Keywords:** traumatic brain injury, coma, EEG, alpha, prognosis

## Abstract

Accurate early prognostication is vital for appropriate long-term care decisions after traumatic brain injury. While measures of resting-state EEG oscillations and their network properties, derived from graph theory, have been shown to provide clinically useful information regarding diagnosis and recovery in patients with chronic disorders of consciousness, little is known about the value of these network measures when calculated from a standard clinical low-density EEG in the acute phase post-injury. To investigate this link, we first validated a set of measures of oscillatory network features between high-density and low-density resting-state EEG in healthy individuals, thus ensuring accurate estimation of underlying cortical function in clinical recordings from patients. Next, we investigated the relationship between these features and the clinical picture and outcome of a group of 18 patients in acute post-traumatic unresponsive states who were not following commands 2 days+ after sedation hold. While the complexity of the alpha network, as indexed by the standard deviation of the participation coefficients, was significantly related to the patients’ clinical picture at the time of EEG, no network features were significantly related to outcome at 3 or 6 months post-injury. Rather, mean relative alpha power across all electrodes improved the accuracy of outcome prediction at 3 months relative to clinical features alone. These results highlight the link between the alpha rhythm and clinical signs of consciousness and suggest the potential for simple measures of resting-state EEG band power to provide a coarse snapshot of brain health for stratification of patients for rehabilitation, therapy and assessments of both covert and overt cognition.

## Teaser

Accurate early prognostication is vital for appropriate long-term care decisions after traumatic brain injury. O’Donnell et al. observe a link between alpha band power in the resting-state electroencephalogram of patients in acute post-traumatic unresponsive states and outcome at 3 months post-injury, indicating the value of electroencephalography for augmenting prognoses.

## Graphical Abstract

**Graphical Abstract:**
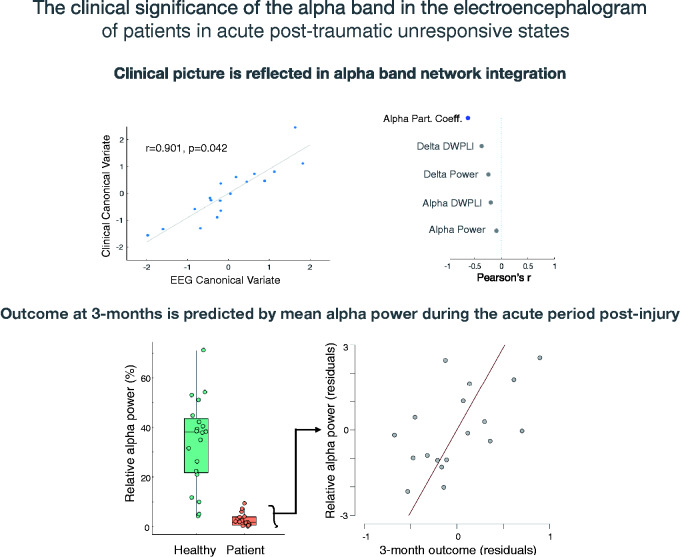

## Introduction

Early and efficient stratification of patients after a traumatic brain injury (TBI) requires accurate prognostication in the intensive care unit. Of those individuals who enter a coma as a result of a TBI, 40% will die in the intensive care unit, 40% will achieve a good recovery and 20% will develop a prolonged disorder of consciousness such as unresponsive wakefulness syndrome (or vegetative state) in which they appear entirely unaware of themselves and their environments.^1^^,^^2^ More accurate prognoses in acute states of unresponsiveness will allow for more appropriate critical care decisions relating to continuation or withdrawal of therapy, thus increasing the effectiveness of public health services and ensuring quality of life for those who progress beyond coma.

Recently, Claassen et al.^3^ demonstrated that a significant minority of patients (16/104) in the acute period after severe brain injury could modulate their EEG-detected brain activity in response to verbal instructions. Furthermore, half of those patients (eight in total) progressed to being able to function independently (i.e. Extended Glasgow Outcome Score (GOSE) ≥4),^4^ compared with a quarter of patients who did not exhibit evidence of instruction-induced EEG modulations. This result builds on evidence of the diagnostic and prognostic utility of task-based EEG modulations in more chronic stages of brain injury.^5^^,^^6^ These task-based approaches identify those patients with the highest level of residual (though covert) cognition and consciousness, thus allowing strong conclusions regarding long-term outcomes. Nevertheless, the evidential ‘bar’ set by task-based approaches limits their sensitivity to those patients who possess the necessary residual cognition to produce appropriate EEG changes. Indeed, EEG changes due to motor imagery—the task employed by Claassen et al.—have low sensitivity in healthy individuals.^7^ Consequently, while the active motor imagery approach allows for strong predictions about future recovery in the 15% of patients who return positive results (i.e. 16/104; see also^8^), there is little clinical benefit for the 85% of patients who return null results.

One approach to address the relative insensitivity of task-based approaches is to use resting-state measures of brain activity, such as band power and functional connectivity. Indeed, resting-state measures from EEG and PET appear to be the most useful for diagnoses in chronic (prolonged) disorders of consciousness.^9^^,^^10^ Evidence also suggests that EEG spectral measures across canonical frequency bands (alpha, theta, and delta) may hold prognostic value for acute post-traumatic coma. For example, alpha power is negatively associated with outcome in early studies^11^ (although see^12^) and, building on these findings, a recent study by Tolonen et al.^13^ demonstrated that alpha band power correlated with clinical outcomes 6–12 months post-injury, as measured by the Glasgow Outcome Scale (GOS). Schnakers et al.^14^ reported a significant positive relationship in a group of 13 patients in acute severe post-TBI coma between alpha power and GOSE at 6 months, which survived statistical controlling for confounds including sedation level. Furthermore, they observed that the weaker the alpha power in the acute stage, the greater the atrophy of the left dorsal and ventral thalamus at 6 months, indicating a link with the known thalamic pathology of prolonged disorders of consciousness^15^^,^^16^. Greater percentage alpha variability (a measure of the daily waxing and waning rhythm of alpha power) has also been demonstrated to predict higher GOS scores at 30 days^17^ and 6 months post-admission,^18^ while other studies have indicated prognostic utility across multiple canonical frequency bands.^19^^,^^20^ Finally, Beridze et al.^21^ observed that an increase in delta band power was associated with progression from coma to unresponsive wakefulness syndrome and death. The correlations between alpha/delta power and positive/negative outcomes, respectively, highlight the utility of these specific measures for prognostic purposes in post-traumatic coma.

Beyond measures of EEG band power, the importance of EEG derived connectivity metrics for prognostication in disorders of consciousness has also been highlighted in recent work. For example, Chennu et al.^22^ identified high-density EEG (hdEEG) as a tool to characterise EEG connectivity networks, using graph theory to extract summary signatures of those networks. In that study, Chennu et al. demonstrated that not only did relative alpha and delta power relate to a patient’s diagnosis, but that the structure of the alpha band connectivity networks—as measured by participation coefficient and modular span (see Materials and methods for definitions)—also related to the patients’ behavioural signs of awareness. Furthermore, in a larger group of patients, Chennu et al.^23^ investigated the prognostic value of network measures in prolonged disorders of consciousness and observed that delta band network modularity and clustering best discriminated good from bad outcome (i.e. death and unresponsive wakefulness syndrome). More recently, Bareham et al.^24^ demonstrated that the accuracy of 3-month outcome predictions could be augmented by the addition of a composite measure of EEG band power, connectivity and graph theory metrics, adding to the body of evidence surrounding the value of EEG in prolonged disorders of consciousness.

Although these graph theory metrics for prognostic purposes appear promising, their utility in the context of acute TBI is yet to be investigated. Nevertheless, and consistent with the potential added value of EEG connectivity measures beyond more conventional measures of spectral power, Kustermann et al.^25^ reported that alpha band connectivity on the first day of post-anoxic coma distinguished between patients with good and bad outcome. We therefore hypothesized that EEG connectivity networks, summarized by graph theory metrics, would predict outcome of patients at 3 and 6 months post-TBI. To test this hypothesis, we recorded resting-state EEG in a group of patients who were entirely sedation free and who had not regained the ability to follow verbal commands in the intensive care unit. Furthermore, we aimed to test the reliability of network measures in the conventional 10/20 EEG recordings made in typical acute care settings. Indeed, while the literature on the clinical value of EEG connectivity networks in chronic patient groups has used high-density recordings (i.e. 64+ electrodes), conventional clinical EEG recordings worldwide are conducted with a low-density montage. We therefore first investigated the reliability of the individual network measures across high- and low-density montages to robustly estimate the underlying cortical dynamics in a group of healthy control participants, before investigating their prognostic utility. This approach ensures confidence that a low-density EEG network measure is a reliable estimate of the same network measure that has shown clinical value in hdEEG of chronic patient groups.

## Materials and methods

### Healthy participant data

We used healthy participant EEG data from the baseline resting-state recordings made for a previous study.^26^ Data from 20 participants with clean EEG data were included in the analyses (11 females; mean age = 30.85; SD = 10.98).

### Patient participants

We screened all 139 TBI admissions to the intensive care unit of the Queen Elizabeth Hospital, Birmingham, UK, between April 2018 and October 2019. Inclusion criteria of this study required patients to have a Glasgow Coma Scale (GCS)^27^ motor score below 6 (i.e. not obeying commands), to be aged over 18 years and to be receiving care as a result of a TBI. Exclusion criteria were: patients moribund, those with prior history of moderate or severe TBI or neurological disorder, those who were not English speakers, those with CT evidence of brainstem-only lesion (i.e. suspected locked-in syndrome), those with CT evidence of focal left lateral temporal lobe lesions (i.e. suspected specific language deficits) and those with known hearing impairments (due to the use of auditory/linguistic stimulation in other aspects of the research protocol).

Of the 139 screened patients, 28 patients were consented onto the study and 21 patients met all inclusion/exclusion criteria at the time of EEG, between 48 h and 7 days after sedation hold. After excluding data from one patient due to technical issues with the recording, 18 patients were available for follow-up at 3 months (median age: 59, range 20–86; 3 females), and 17 patients were available for follow-up at 6 months (median age: 59, range 20–86; 3 females). All patients scored below 6 on the GCS Motor score at the time of EEG—i.e. they did not obey verbal commands (see Table 1 for patient details).

**Table 1:** Demographics and clinical data of the patient cohort

ID	Gender	Age at injury (years)	CT grade	Time since injury (days)	Total GCS at EEG	3-month GOSE	6-month GOSE
1	Male	72	2	5	5	1	1
2	Female	86	2	5	6	1	1
3	Male	26	5	17	6	2	2
4	Male	40	5	12	5	3	3
5	Male	59	5	13	5	3	3
6	Female	44	5	10	6	3	3
7	Male	82	5	3	3	2	3
8	Male	20	6	17	11	4	4
9	Male	70	5	5	6	3	2
10	Male	24	5	24	8	3	4
11	Male	70	6	10	10	6	7
12	Male	27	2	19	7	3	3
13	Male	77	2	12	6	3	3
14	Male	54	2	10	6	6	6
15	Male	59	3	9	6	3	Lost to follow-up
16	Female	59	5	14	8	3	3
17	Male	61	2	15	8	4	6
18	Male	32	2	17	10	4	5

### EEG acquisition and processing

A clinical electrophysiologist recorded the EEG data at 256 or 512 Hz with a 19-electrode clinically certified EEG system, using an XlTek Brain Monitor EEG amplifier (Natus Medical Incorporated, Pleasanton, USA) with a 10/20 montage and additional right and left mastoid electrodes. The ground and reference electrodes were placed across the vertex. Data quality was monitored during acquisition and in subsequent offline artefact correction. We aimed to record between 5 and 10 min of resting-state data per patient, dependent on their level of agitation or immediate care needs.

EEG data were analysed in MATLAB using the MOHAWK toolbox (https://github.com/srivaschennu/MOHAWK).^22^^,^^23^^,^^28^^,^^29^ This toolbox performs the same pre-processing and feature extraction methods employed in previous studies of resting EEG in disorders of consciousness.^22^^,^^23^ Briefly, EEG data are filtered between 0.5 and 45 Hz and segmented into 10-s epochs. A semi-automated combination of visual inspection and independent component analysis removes channels and epochs contaminated by excessive artefact. Finally, the EEG data are re-referenced to the average of all channels. Across patients, a mean of 5.389 min (SD = 1.656) of data was available for analysis.

### EEG feature extraction

We extracted EEG features using the MOHAWK toolbox. Briefly, spectral phase and power of the pre-processed EEG data are estimated in three canonical frequency bands: alpha (8–13 Hz), theta (4–8 Hz) and delta (0–4 Hz) frequency bands. For each frequency band, connectivity between all channel pairs is estimated with the debiased weighted phase lag index.

This matrix of channel-pair connectivity estimates in each frequency band is then submitted to graph theory analyses. Graph theory metrics seek to describe a high-dimensional network as a series of connected nodes. We elected to extract only those graph theory and spectral metrics that have previously been demonstrated to provide diagnostic and/or prognostic information in disorders of consciousness, namely: Relative alpha power (mean over channels), alpha connectivity (median debiased weighted phase lag index over channels), alpha modular span, alpha participation coefficient (standard deviation over channels), relative delta power (mean over channels), delta clustering coefficient (mean over channels) and delta modularity. All graph metrics were averaged over connection density thresholds (90–10%). We also included delta connectivity (median debiased weighted phase lag index over electrodes) in our analyses for symmetry with the alpha features, although removal of this feature does not change our results. ‘Modular span’ describes the average topographical distance spanned by a module in a network. ‘Participation coefficient’ measures the centrality of a network that identifies nodes linking several modules. ‘Clustering coefficient’ describes the local efficiency of nodes in a network. ‘Modularity’ describes the separation of network nodes into isolated modules.

### EEG feature validation

In previous studies using similar graph metrics, hdEEG has been available for each patient (at least 91 channels). However, hdEEG is a challenge in the acute setting due to intracranial pressure monitors, head wounds, etc. Therefore, clinical EEG data are typically recorded in a low-density montage of 19 channels. Therefore, to assess which of the above EEG features can be accurately estimated with this low-density montage, we first pre-processed and extracted EEG features for each of our healthy participants twice: once using data from the 91 high-density channels, and once using only data from the 19 channels of the 10–20 system. Note that we selected the 19 channels before pre-processing, rather than after, to ensure that the data are processed in the exact same manner as the clinical data. Next, we calculated the correlations between each of the EEG measures across montages. The logic here is that those low-density EEG features that significantly correlate with their respective high-density features are those that robustly estimate the underlying cortical dynamics and, therefore, have potential prognostic value.

### Statistical analysis

Following a similar approach to a study of prolonged disorders of consciousness,^24^ we performed a canonical correlation analysis (CCA) to investigate the relationship between EEG features identified from the validation procedure above and the clinical picture at the time of EEG. CCA is beneficial when investigating the relationship between two sets of multiple variables—in our case, the set of clinical variables describing the clinical picture at the time of EEG [age, total GCS score, CT grade (1 = No visible intracranial pathology; 6 = non-evacuated mass lesion^30^), days since injury] and the set of EEG features identified from the validation procedure above. CCA returns canonical variates—linear combinations of variables within each set—that maximally correlate across sets. To test the significance of our canonical variates, we conducted a randomization test with 2000 permutations in which we randomly shuffled the rows (i.e. patients) of the clinical variables relative to the EEG variables, conducted the CCA analysis and recorded the largest correlation between pairs of canonical variates. This approach produces a distribution of 2000 possible correlations between canonical variates under the null hypothesis of no true relationship between patients’ clinical and EEG variables. The *P*-value of the correlation between our true observed canonical variate pair is therefore the proportion of larger correlations observed in the randomization test.

To investigate the prognostic value of the EEG features at 3 and 6 months, we conducted a stepwise linear regression (entry *P* = 0.05, removal *P* = 0.10; using JASP software version 0.12.2^31^) at each follow-up point using GOSE as the dependent variable and the EEG features and five clinical features above as predictors. The GOSE score was normalized using the rank-based inverse Gaussian method prior to analyses to achieve a normal distribution of the dependent variable prior to regression.^32^

### Ethics

The clinical study was approved by the West Midlands Coventry and Warwickshire Research Ethics Committee and the Health Research Authority and was sponsored by the University of Birmingham, England. Personal or Nominated Consultees of each patient were identified by the clinical team and approached to provide written consent. Consultees also consented to be contacted for outcome interviews. Patients who regained capacity during the follow-up period also re-consented. The study was coordinated by the National Institute for Health Research Surgical Reconstruction and Microbiology Research Centre, University Hospitals Birmingham. The results of this study are reported according to the ‘STrengthening the Reporting of OBservational studies in Epidemiology’ statement for reporting observational studies.

### Data availability

All data and analysis scripts are available in the following repository: https://osf.io/8w6qr/.

## Results

### Reliability of EEG features across montages in healthy participants

Of the eight EEG features investigated, five significantly correlated across montages in healthy participants (*P* < 0.05): mean relative alpha power (*r* = 0.988, *P* < 0.001), median alpha connectivity (*r* = 0.958, *P* < 0.001), standard deviation of alpha participation coefficient (rho = 0.489, *P* = 0.030; Shapiro–Wilk test for bivariate normality *P* = 0.031), mean relative delta power (*r* = 0.991, *P* < 0.001) and median delta connectivity (rho = 0.534, *P* = 0.017; Shapiro–Wilk test for bivariate normality *P* < 0.001). We can therefore be confident that these five features, which have previously been linked to diagnosis and prognosis in high-density data from chronic disorders of consciousness, can be reliably estimated from the low-density montages typical of clinical EEG recordings. We therefore selected these five features for investigation in our clinical data below.

### Relationships between EEG features and clinical picture

**Figure 1:**
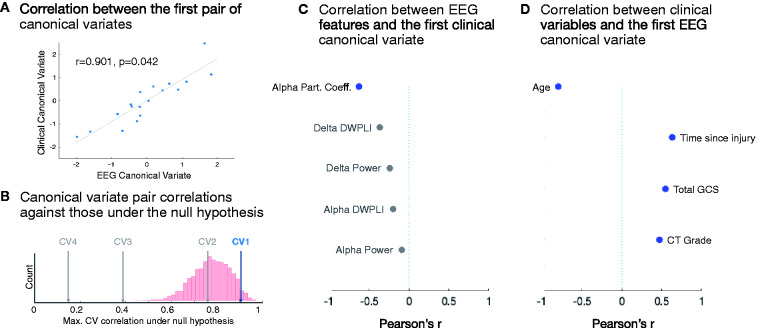
**Relationships between EEG features and clinical picture**. (**A**) Correlation between the first pair of canonical variates, including least squares line fit. *P*-value taken from randomization test of CCA. (**B**) Correlations between each pair of true canonical variates (CVs) against the distribution of maximum CV correlations from the randomization test (i.e. the null hypothesis). CV1 = the CV pair shown in **A**. Non-significant CV pairs are shaded grey. (**C**) Pearson’s correlation coefficients between each individual EEG feature and the first clinical canonical variate. Correlations that were not significant (*P* ≥ 0.05) are shaded grey. (**D**) Pearson’s correlation coefficients between each individual clinical variable and the first EEG canonical variate.

Our CCA approach identified a significant correlation between the first pair of clinical and EEG canonical variates (*r* = 0.901, randomization test *P* = 0.042; Fig. 1A). All other pairs of canonical variates were non-significant in the randomization test (all *P* > 0.561; Fig. 1B). The first EEG canonical variate significantly correlated with all clinical variables (Fig. 1D) while the first clinical canonical variate significantly correlated with the standard deviation of alpha participation coefficient only (*r* = −0.622, *P* = 0.007; Fig. 1C).

### EEG features for prognostication

**Figure 2:**
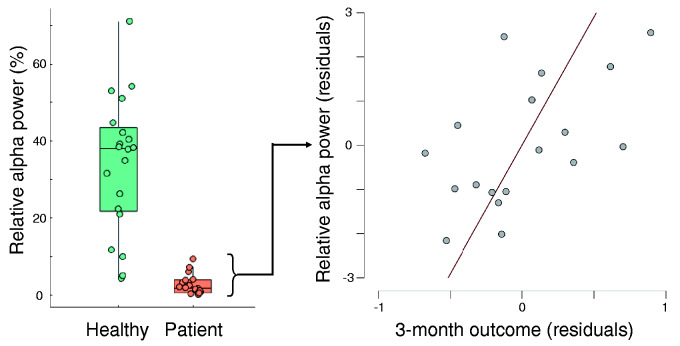
**The prognostic value of relative alpha power**. (Left) Tukey boxplot of relative alpha power for the healthy control group and the patient group. (Right) Residuals scatter plot of relationship between relative alpha power (mean across electrodes) and outcome at 3 months.

The adjusted variance of outcome at 3 months was best explained by a model containing GCS at the time of EEG (beta = 0.684, *P* = 0.001) and mean relative alpha power [beta = 0.389; *P* = 0.039; adjusted *R*^2^ = 0.502, *F*(2,15) = 9.580, *P* = 0.002]. This model explained significantly more variance of outcome at 3 months than a model containing GCS at the time of EEG alone (*R*^2^ change = 0.150) indicating the added value of mean relative alpha power beyond behavioural measures alone (see Fig. 2). At 6 months, the adjusted variance of outcome was best explained by a model containing only GCS at the time of EEG [beta = 0.590, *P* = 0.013; adjusted *R*^2^ = 0.305, *F*(1,14) = 8.018, *P* = 0.013].

## Discussion

The relative preservation of resting-state EEG oscillatory networks has been shown to provide diagnostic and prognostic information for prolonged disorders of consciousness.^9^^,^^22^^,^^23^ Here, we tested the hypothesis that similar network measures in clinically-standard resting-state EEG recordings have prognostic value for acute post-traumatic unresponsive states. While our results indicate a valuable relationship between resting-state EEG oscillations and outcome at 3 months post-injury, we find no evidence for the prognostic utility of network measures in this data.

Our primary finding is that relative alpha power, averaged across the head during the acute stages of injury, provides prognostic information beyond that provided by conventional clinical measures for prognosis at 3 months. Importantly, this link is evident for those patients who remain unresponsive to verbal commands after washout of sedation, and who are therefore otherwise in positions of greatest prognostic uncertainty.

The lack of evidence for prognostic value of network measures may reflect our low-density scalp coverage. Indeed, graph theory measures of EEG frequency band connectivity networks are valuable as summary descriptors of high-dimensional relationships. It is possible that the low-density coverage of the standard clinical 10/20 EEG is not sufficiently high-dimensional for graph theory metrics to capture relationships that cannot otherwise be estimated from the signal itself. Indeed, of the graph metrics we considered, only one (SD of alpha participation coefficient) could be significantly estimated from low-density recordings in healthy participants, and its relationship was the weakest of all other significant measures (rho = 0.489, *P* = 0.030). While our current data cannot address this, it is further possible that the cross-montage validity of graph metrics varies across patient groups and/or levels of consciousness, thus obscuring their prognostic value here. Nevertheless, a simple measure of relative alpha power averaged across the head augments prognostic accuracy at 3 months post-injury. Indeed, in our comparison of low-density and high-density estimates in healthy participants, mean relative alpha power is highly consistent regardless of the level of scalp coverage, with a correlation coefficient of 0.988, thus indicating its utility across EEG provision.

Similarly, the average length of our EEG data (∼5 min) was somewhat lower than the ≥10 min used in previous studies,^22–24^ which may have contributed to the decreased efficacy of the graph theory metrics. However, in a *post*  *hoc* analysis, we calculated our graph theory measures of interest from both 10 and 5 min of healthy control hdEEG data and found large and highly significant correlations between recording lengths for all measures (all *r* ≥ 0.800, all *P* < 0.001; see ‘Data Repository’), giving us confidence in the robustness of graph theory metrics in 5-min hdEEG recordings.

The prognostic importance of relative alpha power is consistent with the mesocircuit hypothesis,^33^ which proposes that alpha activity reflects the intact functioning of thalamo-cortical loops. These loops are considered a prerequisite for consciousness.^33^^,^^34^ When they are not functional, for example due to thalamic damage or inhibition caused by reduced background neuronal activity, consciousness is impossible.^35^ The presence of alpha activity thus suggests that connections in the thalamo-cortical loop are intact, and future recovery of consciousness is possible.^33^ The mesocircuit hypothesis is supported by the observation that damage to the thalamus is very common in disorders of consciousness, and the level of damage is greater in unresponsive wakefulness syndrome compared to the minimally conscious state.^15^ The sedative Zolpidem, which indirectly causes disinhibition of the thalamus via inhibition of the globus pallidus, also increases the level of consciousness in some patients with disorders of consciousness,^35–38^ and there is some evidence that electrical stimulation of the thalamus increases patient responsiveness.^39^ In summary, alpha activity may reflect the necessary structural and functional connections required for the brain to support consciousness in the future, despite the apparent absence of consciousness at the time of the EEG recording.

A second, complementary, hypothesis is that alpha activity shapes the contents of consciousness.^40^ Jensen and Mazaheri^41^ propose that alpha oscillations play an active role in perception and cognition by inhibiting task-irrelevant processing. In support of this hypothesis, alpha power is observed to increase in the primary sensory cortices of task-irrelevant sensory modalities.^41^ Pre-stimulus alpha activity in a motor task is also negatively associated with performance, perhaps predicting attentional lapses before they occur.^42^ This inhibition appears phasic, with periods of inhibition enhancing perceptual abilities at certain phases of alpha activity.^41^^,^^43^^,^^44^ It follows from this hypothesis that the relatively preserved alpha rhythms evident in some patients may reflect conscious contents at the time of EEG recording. However, the data do not allow us to draw this conclusion as it requires a significant reverse inference, and because we have no corroborating evidence of the covert conscious state of the patients. Future studies that combine results of both passive and active measures are required to delineate the functional significance of alpha network activity in non-responsive patients.

Our lack of evidence that EEG features contribute to the accuracy of outcome prediction at 6 months, despite being relevant for 3-month outcome prediction, raises an interesting possibility regarding EEG-based methods for prognostication after severe brain injury. Indeed, Claassen et al.^3^ observed a relationship between 12-month outcome and acute EEG evidence of cognitive–motor dissociation—i.e. a high level of complex cognition indicative of residual, but undetected, consciousness. While we do not have sufficient 12-month outcome data for our cohort, the evidence that the value of acute resting-state EEG markers reduces over time (from 3 to 6 months) suggests that the two types of EEG approach—passive and active—characterize distinct and complementary aspects of potential for recovery. A resting-state EEG may provide a coarse snapshot of residual brain health, including the level of acute damage within the mesocircuit, and thereby indicate the relative preservation of the necessary neural precursors to consciousness in the future. Nevertheless, evidence of these precursors alone is insufficient at longer timescales, as some, but not all, patients’ recoveries build upon those neural precursors. An active command-following EEG, on the other hand, indicates acute preservation of the cortical networks that actively support the contents of consciousness, and so may be more robust to longitudinal variation. A regular schedule of resting-state EEGs after severe brain injury may allow for a richer picture for long-term prognosis on the basis of multiple coarse snapshots of residual brain health. Indeed, Bareham et al.^24^ reported that the most accurate prognoses in prolonged disorders of consciousness were derived from the rate of change between a set of EEG metrics acquired 3 months apart. A similar approach in the acute period may be just as beneficial.

A more fine-grained assessment of the level of recovery achieved by each patient in our study may also have revealed further prognostic relationships, or helped in interpreting the relative value of our approach at longer outcome time-points (i.e. 6 versus 3 months). While we quantified outcome using a standardized telephone interview assessment tool (GOSE), in-person tools for differential diagnosis of disorders of consciousness, such as the Coma Recovery Scale—Revised,^45^ may have increased the sensitivity of our outcome measures and allowed for more detailed characterizations of each patient. Indeed, future research could also use the Coma Recovery Scale—Revised to quantify each patient’s abilities at the acute stage, thus allowing direct investigation of longitudinal behavioural changes within the same scale, as has shown value in prior work.^24^

A further consideration for the clinical application of EEG for prognosis is the relative sensitivity of active and passive approaches. As noted above, an active motor imagery approach is of benefit to those patients who return a positive result—∼15% of patients^3^^,^^8^—while a passive approach can provide a continuous measure of relative cortical function in the vast majority of patients. Indeed, in our study, only one patient’s EEG recording was too noisy to be analysed. Consequently, one could foresee a hierarchical approach to EEG-based prognostication after severe brain injury, with active paradigms targeted to those patients who exhibit relative alpha functional preservation in an initial resting-state recording. Indeed, as motor imagery is primarily reflected in modulations of alpha (or mu) rhythms,^46^ a patient with a residual resting alpha rhythm may be more likely to have top-down control over that rhythm to complete active motor imagery tasks and demonstrate their cognitive-motor dissociation.

Our CCA approach revealed a relationship between the first EEG canonical variate and patients’ clinical picture at the time of EEG. Specifically, the EEG canonical variate correlated negatively with patients’ age at the time of injury and positively with their total GCS score at the time of EEG, their CT grade and the time passed between injury and EEG. This result validates the view that a snapshot EEG provides information relating to the current clinical picture of the patient. Conversely, the clinical canonical variate negatively correlated with only the standard deviation of alpha participation coefficient. The participation coefficient is a measure of network centrality that highlights hub nodes that link modules within the network, with large standard deviations across nodes (i.e. electrodes) indicative of more integrated networks.^23^ The standard deviation of participation coefficients in the alpha network has previously been shown to increase with increasing behavioural signs of awareness,^23^ to differentiate those unresponsive patients with evidence of covert awareness from those without^22^ and to differentiate between patients with and without relatively preserved PET-detected frontoparietal metabolism.^10^^,^^23^ Our observation of a link between this measure of alpha band network complexity and patients’ acute clinical picture after severe brain injury is therefore consistent with these previous links between alpha band network complexity and the relative preservation of behavioural responsiveness and the neural foundations of awareness in chronic disorders after severe brain injury. Indeed, integrated brain networks are considered to be a necessary condition for consciousness according to several key theories.^47^^,^^48^ Despite this, alpha power, rather than participation coefficient, was most strongly linked to patient outcome, perhaps due to the low statistical power of graph metrics in low-density montages.

In conclusion, a growing body of evidence links the EEG alpha rhythm with both levels and contents of consciousness in the clinic. While we did not find evidence that more complex measures of alpha network features provide prognostic information in standard low-density clinical EEG recordings, our evidence for a link between mean relative alpha power and outcome at 3 months suggests the potential for a simple and standard EEG measure to augment prognostication in post-traumatic states of unresponsiveness. This approach may provide a coarse snapshot of brain health for stratification of patients for rehabilitation, therapy and subsequent fine-grained assessments of both covert and overt cognition.

## Abbreviations

CCA =: canonical correlation analysis;
hdEEG =: high-density EEG;
GCS =: Glasgow Coma Scale;
GOSE =: Glasgow Outcome Score Extended;
TBI =: traumatic brain injury
